# From COVID-19 to Sarcoidosis: How Similar Are These Two Diseases?

**DOI:** 10.3389/fimmu.2022.877303

**Published:** 2022-05-09

**Authors:** Min Zhao, Chang Tian, Shan Cong, Xin Di, Ke Wang

**Affiliations:** Department of Respiratory and Critical Care Medicine, The Second Hospital of Jilin University, Changchun, China

**Keywords:** COVID-19, sarcoidosis, granulomatous disease, renin–angiotensin system, cell death pathways

## Abstract

Coronavirus disease 2019 (COVID-19), which is caused by severe acute respiratory syndrome coronavirus 2 (SARS-CoV-2), leads to the dysregulation of the immune system, exacerbates inflammatory responses, and even causes multiple organ dysfunction syndrome in patients with severe disease. Sarcoidosis is an idiopathic granulomatous multisystem disease characterized by dense epithelioid non-necrotizing lesions with varying degrees of lymphocytic inflammation. These two diseases have similar clinical manifestations and may also influence each other and affect their clinical courses. In this study, we analyzed some possible connections between sarcoidosis and COVID-19, including the role of the renin–angiotensin system in the respiratory system, immune response, and cell death pathways, to understand the underlying mechanisms of SARS-CoV-2 infection, predisposing patients to severe forms of COVID-19. This review will provide a new prospect for the treatment of COVID-19 and an opportunity to explore the pathogenesis and development of sarcoidosis.

## Introduction

Severe acute respiratory syndrome coronavirus 2 (SARS-CoV-2) is a novel virus that was first detected in December 2019 as an etiological agent in a group of pneumonia patients. SARS-CoV-2 is responsible for the coronavirus disease 2019 (COVID-19) pandemic. According to published data statistics, most of the SARS-CoV-2 infection-related deaths occurred in patients with comorbidities and those above 60 years of age (1–3). Patients with autoimmune diseases have increased chances of microbial infection (4) because they have a potentially unstable immune system, which is caused by the frequent administration of oral immunosuppressants (5).

Sarcoidosis is an idiopathic granulomatous multisystem disease characterized by dense epithelioid non-necrotizing lesions with varying degrees of lymphocytic inflammation (6). Although the pathogenesis of sarcoidosis is unclear, it is generally accepted that sarcoidosis is caused by abnormal immune responses in genetically susceptible individuals exposed to specific environmental factors. The majority of sarcoidosis patients require immunosuppressive therapies, which may include corticosteroids such as prednisone (7). People with sarcoidosis have certain characteristics that may increase the risk of SARS-CoV-2 infection and lead to severe COVID-19. Brito-Zerón et al. (8) analyzed the clinical characteristics and outcomes of COVID-19 in sarcoidosis patients in one of the largest multicenter clinical cohorts of sarcoidosis and reported a 5.1% frequency of SARS-CoV-2, and a third of SARS-CoV-2-infected patients with sarcoidosis required hospitalization, with a 9% overall mortality rate. Moreover, patients with moderate and/or severe pulmonary impairment had a higher risk of intubation and mechanical ventilation, leading to higher in-hospital mortality (9). However, O’Driscoll et al. reported that the frequency of SARS-CoV-2 infection with sarcoidosis was similar in 45 countries and regions, with approximately 5% of the population infected under the age of 65 years (10). The renin–angiotensin system (RAS); angiotensin-converting enzyme (ACE), which is present in high levels in the serum of patients with sarcoidosis; and ACE2, which is an essential and major receptor for the SARS-CoV-2 to enter the cell, may also play a key role in the link between COVID-19 and sarcoidosis (11, 12).

Multiple SARS-CoV-2-infected patients have been reported to develop subcutaneous nodules with granulomatous histology similar to sarcoidosis, indicating a potential link between SARS-CoV-2 and sarcoidosis (13–15). Five patients that were reported were all women, and primary sarcoidosis is ruled out. Sarcoidal granulomas mimicking scar sarcoidosis, including subcutaneous nodules on the arms, shins, lateral thighs, glabella, and submental, appeared after 1–2 weeks of a positive reverse transcriptase real-time polymerase chain reaction (RT-PCR) result for SARS-CoV-2 infection. Moreover, cervical lymphadenopathies and pulmonary radiological scans showed pneumonia-like acute inflammatory interstitial lung disease. In the biopsy of three patients, non-caseating granulomas, indicative of sarcoidosis, were detected in the subcutaneous nodular tissue. The occurrence of granulomatous disease in some COVID-19 patients raises some concerns, as both diseases could be linked not only in clinical manifestations but also in etiology.

**Figure 1:**
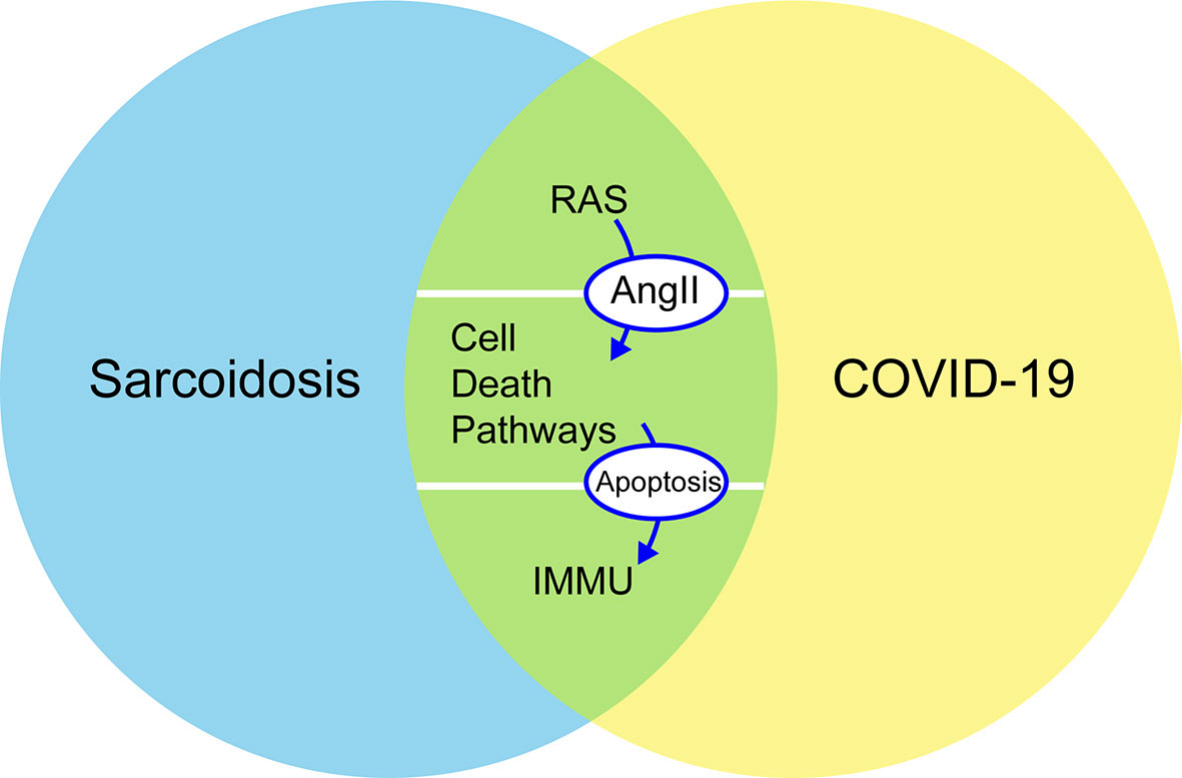
Common mechanistic of COVID-19 and sarcoidosis including RAS, immune responses, and cellular death pathways (autophagy, apoptosis, and PD-1/PD-L1 axis): increased concentrations of Ang II stimulate apoptosis and are negative regulators of autophagy. PD-1 increases the apoptosis of specific T cells in lymph nodes and decreases the apoptosis of regulatory T cells. RAS, renin–angiotensin system; Ang II, angiotensin II; PD-1, programmed cell death 1; IMMU, immune responses.

To better understand the interaction between sarcoidosis and COVID-19 in terms of clinical manifestations, treatment, and pathogenesis, this study discusses how the two diseases might involve some common mechanistic immune responses, including RAS in pulmonary and some cell death pathways around the regulation of autophagy, apoptosis, and programmed cell death (PD-1/PD-L1 axis) (**Figure 1**). The objective of this review was to analyze some common characteristics of sarcoidosis and COVID-19 to improve our understanding of the underlying mechanisms predisposing patients to severe COVID-19 and the pathogenesis of sarcoidosis and to find effective treatments during the COVID-19 pandemic.

## RAS and Its Inhibitors

### The Renin–Angiotensin System

**Figure 2:**
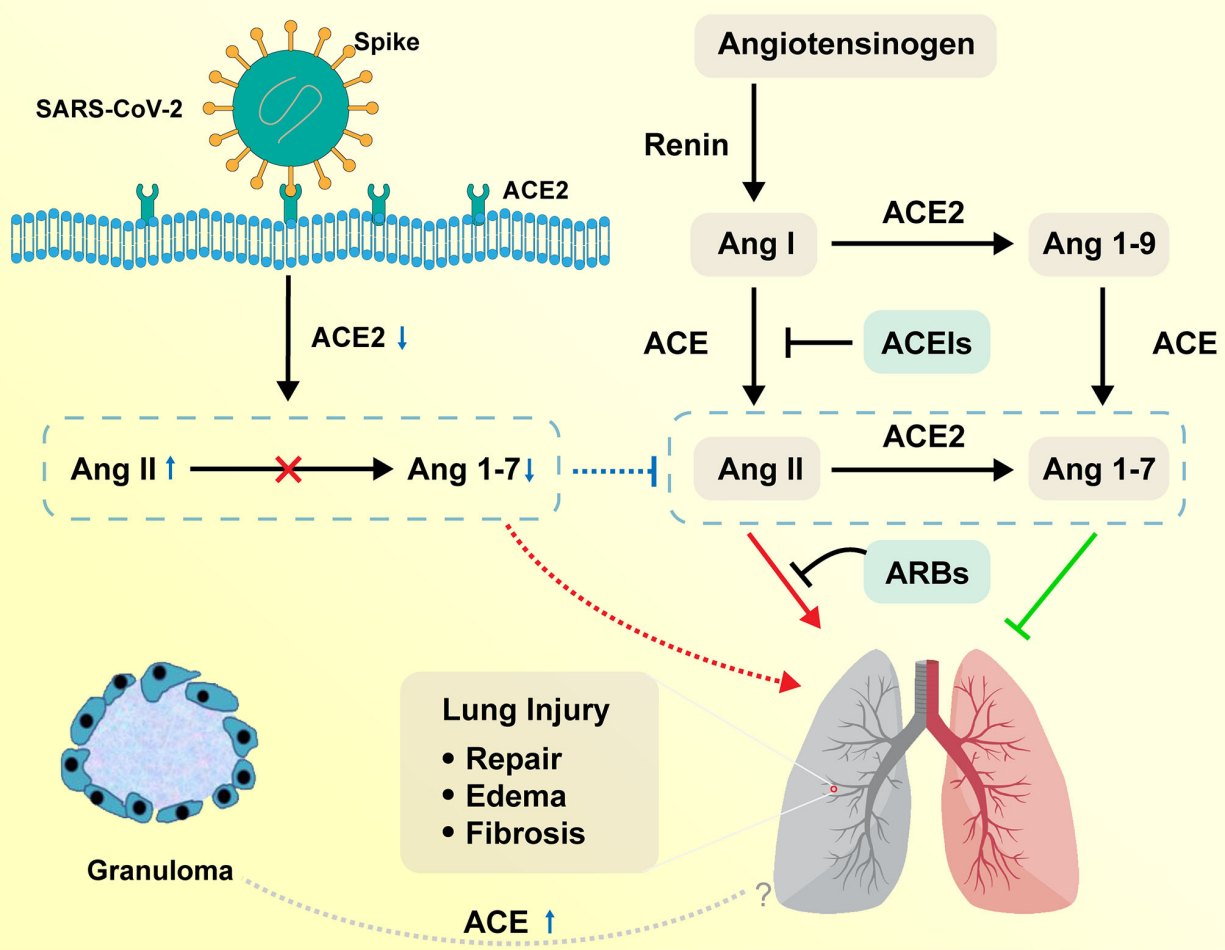
Function of RAS in the respiratory system on COVID-19 and sarcoidosis. Angiotensinogen is converted by renin to Ang I. Ang I is subsequently converted to Ang II by ACE, which is expressed on the surface of endothelial cells in the lung and kidney. Ang II in the pulmonary system promotes tissue repair and fibrosis, while it can also promote the occurrence of pulmonary edema and impair lung function. ACE2 negatively regulates the function of ACE by converting Ang I to Ang 1–9 and Ang II to Ang 1–7. ACEIs inhibit the production of Ang II and ARBs inhibit the binding of Ang II to angiotensin receptors. SARS-CoV-2 interacts with ACE2 and infects ACE2-expressing epithelial and endothelial cells in the lung and other organs, leading to the downregulation of ACE2. The downregulation of ACE2 leads to unopposed Ang II accumulation, which may accelerate the progress of COVID-19 *via* increased activity of RAS. Sarcoidosis granuloma epithelioid cells may be engaged in ACE biosynthesis, resulting in the elevation of serum ACE levels. The relationship between elevated ACE and sarcoidosis remains to be explored. RAS, renin–angiotensin system; ACE, angiotensin-converting enzyme; Ang I, angiotensin I; ACEIs, angiotensin-converting enzyme inhibitors; ARBs, angiotensin receptor blockers.

The homeostasis regulation system relies on RAS as a mediator (16). The ACE, angiotensin II, and angiotensin II type 1 (ACE–Ang II–AT1R) pathway is called a classical RAS axis that helps maintain homeostasis by facilitating dynamic balancing of cardiovascular function (17). The negative regulatory axis mediated by ACE2, which is a homolog of ACE and was first reported in 2000 (18), can antagonize these effects. The lungs have high RAS activity, including the synthesis of ACE, and are the leading site of Ang II synthesis, i.e., local RAS signaling (19, 20). RAS is activated in the lungs after a pulmonary injury to promote pulmonary repair, but when in excess, it promotes tissue edema, leads to pulmonary fibrosis, and further impairs pulmonary function (21, 22). On the contrary, ACE2 acts as a negative regulator of the RAS by inactivating Ang II and producing Ang 1–7, which exhibit vasodilatory, anti-inflammatory, and antifibrotic effects (23, 24). Higher Ang II/Ang 1–7 ratio favors increased pulmonary vascular permeability and further leads to the accumulation of extra-alveolar fluid (**Figure 2**) (25).

The tropism of SARS-CoV-2 to the respiratory system is maintained by the binding of SARS-CoV-2 to the ACE2 receptor on human alveolar epithelial cells (26). ACE2 is widely expressed in human alveolar epithelial cells, particularly alveolar type II epithelial cells (27–29). The binding of the ACE2 receptor to the transmembrane spike (S) glycoprotein, which takes the shape of homotrimers protruding from the surface of the coronavirus, mediates the entry of SARS-CoV-2 into human body host cells (30).

Following the outbreak of SARS in 2003, ACE2 was found to be the receptor in the respiratory system that allows SARS-CoV to enter the human body, and was proven to be the activation site of the disease (31). It is believed that SARS‐CoV and SARS‐CoV-2 share similar pathogenesis and pathology (32). Some severe COVID-19 and SARS patients were reported to have developed typical acute respiratory distress syndrome (ARDS), which is a clinically rare and severe acute lung injury with high mortality (17). In a clinical cohort analysis, the plasma Ang II levels in SARS‐CoV‐2-infected patients were significantly higher than those in healthy individuals (33). However, researchers found that SARS‐CoV‐infected patients exhibited significantly reduced ACE2 expression in the lungs. As a result, the reduced expression of ACE2 may have a causative role in the pathogenesis of SARS, providing a reasonable explanation for the progression of ARDS in COVID-19 and SARS patients (34). Further experiments showed significant downregulation of the ACE2 protein expression in an ARDS mouse model and reported that AT1R-meditated upregulation of Ang II expression plays an important role in acute lung injury (35). Imai et al. (36) have confirmed that ACE2 and AT2R protect mice from severe acute lung injury caused by acid aspiration or sepsis and that exogenous recombinant human ACE2 reduces plasma Ang II levels and the severity of acute lung failure in Ace2 knockout mice. Studies that monitored mouse models with acute lung injury showed that tracheal instillation of cigarette smoke (37) or particulate matter with an aerodynamic diameter less than 2.5 µm in mouse models (38) induced increased release of inflammatory cytokines, including IL-6, tumor necrosis factor (TNF)-α, and TGF-β1, and upregulation of ACE expression, which was consistent with hyperactivity of the ACE→Ang II→AT1R axis. It has been speculated that overactivation of RAS promotes inflammatory responses and cytokine storms (39), stimulates the NADH/NADPH oxidase system (40), and triggers cellular death and vasoconstriction, which may directly or indirectly contribute to COVID-19-related lung injuries (41). In contrast, pneumocytes type II, where ACE2 is mainly expressed, produce alveolar surfactant and can also be transformed into pneumocytes type I, which are primarily responsible for gas exchange (42, 43). Injured pneumocytes type II reduce alveolar surfactant production and pneumocytes type I repair, leading to impaired gas exchange, lung function, and pulmonary fibrosis. In summary, ACE2 receptor-mediated entry of the coronavirus into the human body causes severe damage to the respiratory system (44).

ACE, an integral membrane-bound protein, is strongly expressed in many types of endothelial cells, including the respiratory capillary endothelial cells (45). It was first reported in 1975 when serum ACE levels were observed to increase in active sarcoidosis patients (46). The possibility of elevated ACE levels in sarcoidosis was explained by the fact that granulomas of epithelioid cells may be primarily involved in ACE biosynthesis rather than phagocytosis and catabolism (47). Sarcoidosis granulomas are formed when the precursor cells are transformed into granuloma cells through a series of cell signaling events stimulated by cytokines. When viral DNA is integrated into the host chromosome, the persistence of viral plasmids or chromosomal mutations may cause granuloma cells to actively synthesize ACE and other cellular molecules, altering the cellular microenvironment surrounding the granuloma (47). There is still no clear evidence that these microorganisms are potential factors in the etiology of sarcoidosis. However, it is unknown if human coronavirus (HCoV), including SARS‐CoV‐2, can induce granuloma formation and increase ACE2 levels.

The clinical course of sarcoidosis varies widely. In a majority of clinical cases, the abnormality of the lung parenchyma observed on lung imaging usually resolves spontaneously, but a minority (approximately 20%–25%) of cases progressively develop pulmonary fibrosis (48). In addition, the degree of pulmonary fibrosis varies from mild and slowly progressive to life-threatening and poor prognosis. The information about the mechanisms that drive spontaneous resolution or persistence of sarcoidal granulomas and their progression to pulmonary fibrosis is limited (7, 48). In the respiratory system, tissue injuries activate lung fibroblasts, and then injured epithelial cells and fibroblasts express RAS components, especially angiotensinogen. Ang II may play a role in regulating cell growth and fibrogenesis as a growth factor (21). However, the relationship between elevated ACE and Ang II in sarcoidosis remains to be explored. Further research is required to determine if high ACE content in sarcoidosis induces the expression level of Ang II and further affects sarcoidosis patients infected with SARS-CoV-2, leading to the progression of sarcoidosis.

### RAS Inhibition

RAS inhibitors mainly include angiotensin-converting enzyme inhibitors (ACEIs) and angiotensin II receptor blockers (ARBs), both of which are used as first-line treatments for hypertension and kidney diseases (49, 50). However, in actual clinical practice, it is unclear whether hypertension patients with SARS-CoV-2 infection and/or combined with sarcoidosis should continue using ACEI/ARB.

RAS inhibition may represent a double-edged sword (51). ACEIs and ARBs directly increase ACE2 expression or mRNA activity (52–54). As a result, the risk of increased susceptibility to infection linked to RAS inhibition introduces more viral entry, which ACE2 expression incurred (55), leading to potential lung damage in COVID-19 patients (34). According to clinical statistics, the rate of COVID-19-related mortality was higher in patients with hypertension, diabetes, and chronic cardiovascular disease, and ACE2 expression was significantly increased in patients with diabetes and hypertension after ACEI/ARB treatment. Fang et al. (56) postulated that elevated ACE2 levels would promote SARS-CoV-2 infection and that ACEI and ARB may further induce disease progression and increase mortality.

Owing to the multiple functions of RAS components in humans, it has been suggested that ACEI and ARB may also play an important role in disease progression (57). Increased ACE2 expression inhibits RAS activity and modulates immune response activity, further potentially serving as a useful tool in the treatment or prevention of pneumonia and ARDS. In a multicenter retrospective study of SARS-CoV-2 infection in hypertensive patients, patients who received ACEIs and ARBs had lower all-cause mortality and septic shock than patients who did not (58). Moreover, Ozbalci (59) pointed out that ACEI and ARB treatment can prevent severe interstitial pneumonia by modulating immune activity, including increasing CD4^+^ T-cell count, improving CD4/CD8 ratio imbalance, and downregulating IL-8. ACEIs and ARBs have been reported to have a significant influence on the occurrence and development of COVID-19 and to promote antibody production (60–62). These data support the American Heart Association’s recommendation against discontinuing ACEIs and ARBs based on the current evidence with COVID-19 patients (55).

Although ACE expression in the serum of sarcoidosis patients is affected by many factors, its sensitivity and specificity are low, and there are genetic polymorphisms in its expression in different populations. Moreover, ACE is still the first and only biomarker for the diagnosis and follow-up of sarcoidosis mentioned in the WASOG sarcoidosis guidelines (63). However, the diagnostic utility of ACE is more doubtful in sarcoidosis patients treated with ACEIs (64, 65). A retrospective study analyzing the changes in serum ACE levels in patients with sarcoidosis and heart disease found that ACE levels in patients treated with ACEIs were significantly and persistently lower than those in patients not treated with ACEIs, suggesting the ability of ACE as a potential biomarker (66).

Experimental studies have shown that ACE is upregulated in macrophages inside granulomas and that inhibiting the level of ACE in *Schistosoma mansoni* granulomas reduces the size of granulomas, indicating that ACE plays a potential role in the formation and maintenance of granulomas (67). At present, there are few reports on the treatment of ACEIs and ARBs for sarcoidosis. It is unclear whether administering ACEIs and ARBs to patients with sarcoidosis has any beneficial effects, including reducing the possibility of pulmonary fibrosis by reducing the level of Ang II or immunomodulatory activity. Further experimental research is required to understand the relationship between ACEIs/ARBs and COVID-19 or sarcoidosis.

## Immune System

Sarcoidosis is pathologically characterized by non-caseating granulomas (68, 69) surrounded by numerous immune cells, including CD4^+^ and CD8^+^ T lymphocytes (70) and a small number of B lymphocytes. The mechanisms underlying the formation, maintenance, progression, and spontaneous resolution of granuloma are unknown (71, 72). In part, sarcoidosis represents a currently unclear theory, the immunological paradox, in which a state of immune anergy around the organism is observed despite an excessive immunoinflammatory response at the site of the granuloma (73), manifested as a suppressed immune response to tuberculin in patients with sarcoidosis (74).

SARS-CoV-2 induces a series of immunoinflammatory responses after entering the human body. In severe cases, it can develop into ARDS and even multiple organ dysfunction syndrome (MODS). Biopsy of lung samples from patients with COVID-19 showed inflammatory cell clusters, including multinucleated giant cells (MGCs) and CD4^+^ T lymphocytes, suggesting a possible association with sarcoidosis, which also has MGCs (75). Some COVID-19 convalescent patients also showed granulomatous manifestations resembling sarcoidosis. Hematological analysis of COVID-19 patients has been reported to show lymphopenia, which is an important indicator of severe SARS-CoV-2 infection and poor prognosis (76–78), suggesting a defect in their adaptive immune system. Multiple sets of clinical data show that the absolute number of circulating lymphocytes is significantly lower in COVID-19 patients in the ICU than in patients with mild to moderate infection (79). However, the immune mechanism in COVID-19 patients is complex and still unclear, especially about potentially orchestrated acute mortality. There are some similar immune response pathways in both sarcoidosis and COVID-19 (**Table 1**).

**Table 1:** The characteristics of sarcoidosis and COVID-19 on the immune system.

		Common	Sarcoidosis	COVID-19
Th1	Lymphocytes	Blood lymphopenia	Peripheral anergy	Related to severe–critical patients
		CD4/CD8 ratio in BALF	CD4/CD8 >3.5	CD4/CD8 >1.5
	Th17	Inflammation-driven polarization into Th1	Th17.1 (produces only IFN-γ)	Th17 cells characterized by GM-CSF production
	Molecular phenotypes	Related to the different phenotypes	LS: co-expression T-bet and ROR-γt T cells Non-LS: both co-expression CXCR3 and CCR6 T cells	Severe patients: GM-CSF and IL-6^+^ co-expression T cells ICU patients: co-expressing IFN-γ and GM-CSF T cells
	IFN	Changes in the numbers	Extensive type II IFN (IFN-γ)	The imbalance of IFN: high levels of IFN-γ and impaired type I IFN response (IFN-α/β)
Treg		Enhanced the inflammatory reactions and granuloma formation	CD45RO^+^CD7^−^ late memory phenotype Treg	The loss of CD45RA^+^ Tregs
Tfh		The alteration of the humoral immune response	B cells: activated naive phenotype and hypergammaglobulinemia	Maturation of dysfunctional B cells
Cytokine storm		Upregulation of a series of cytokine levels	IFN-γ, TNF, IL-12, IL-18 and IL-6, TGF-β and IL-10, IL-2, IL-4 and IL-17A	IL-1β, IL-1RA, IL-2, IL-4, IL-6, IL-7, IL-10, IL-19, G-CSF, TNF-α, IFN-γ

BALF, bronchoalveolar lavage fluid; GM-CSF, granulocyte–macrophage colony-stimulating factor; ROR-γt, retinoic acid receptor-related orphan receptor-γt; LS, Löfgren’s syndrome; Tregs, regulatory T cells.

### Th Cells

An increased number of CD3^+^CD4^+^ T lymphocyte cells was observed in bronchoalveolar lavage fluid (BALF) and other affected tissues of pulmonary sarcoidosis patients, resulting in an increased CD4/CD8 ratio, considerably higher than 3.5. Sarcoidosis patients had lower peripheral percentages of T cells than healthy controls (80). Further experimental research is required to understand if the peripheral anergy and blood lymphopenia of sarcoidosis can induce bacterial or viral infections, including SARS-CoV-2.

It was observed that BALF cell count analysis of hospitalized COVID-19 patients showed an accumulation of CD4^+^ and CD8^+^ T cells (81), similar to sarcoidosis patients, and most cell counts showed CD4/CD8 ratios higher than 1.5, which may explain the reason for the higher decrease in CD8^+^ T-cell number than CD4^+^ T-cell number (82). Autopsy of lung specimens from dead COVID-19 patients also showed varying degrees of interstitial and perivascular lymphocytic infiltration (83). However, the absolute number of peripheral circulating T cells was significantly reduced in COVID-19 patients, which may be because of increased lymphocytosis locally recruited to lung tissue or increased lymphocyte adhesion to the endothelium (84). Moreover, elevated levels of cytokines IL-6, IL-10, and TNF-α were observed in severe COVID-19 patients, directly affecting T-cell subsets and indirectly affecting dendritic cells and neutrophils, thereby reducing lymphocyte count (79, 85).

As additional T-cell subsets were identified with highly accurate discriminating subsets of Th cells, several researchers have found that Th17.1 cells, a large subpopulation of Th17, produce only IFN-γ. Furthermore, in BALF cell counts from sarcoidosis patients, Th17.1 cells were high in number than traditional Th1 cells (86, 87). Studies have shown that Th17 cells are classically polarized plastic cells that can be polarized to differentiate into a Th1-like cell phenotype that produces IFN-γ (88). The lungs of sarcoidosis patients accumulate a large amount of IFN-γ, which can convert Th17 cells into cells with Th1 cell characteristics (89–91). The same was observed in COVID-19 patients, where one subset of Th17 cells exhibiting a tissue residency phenotype and characterized by GM-CSF production was detected in BALF samples. Interestingly, this Th17 subset shares a molecular signature with Th1, suggesting that Th17 cells are polarized into Th1 cell types driven by inflammatory factors at the site of infection (92, 93).

Molecular phenotypes of the immune cells were found to be related to the severity of the disease. Different T-cell subsets have been found to be associated with different types of sarcoidosis. The count of T cells expressing both T-bet and retinoic acid receptor-related orphan receptor-γt (RoR-γt) was significantly increased in the lungs of patients with Löfgren’s syndrome (LS), a type of sarcoidosis associated with a good prognosis for clear disease (94). Additionally, a subset of T cells co-expressing CXCR3 and CCR6 was observed in the BALF and lymph nodes of non-LS patients and was associated with disease progression. The role of different T-cell subsets in sarcoidosis needs to be further discussed (95). Meanwhile, higher numbers of CD4^+^ T-cell subtypes expressing GM-CSF and IL-6^+^ were observed in COVID-19 patients in the ICU with more severe pneumonia. Aberrant Th1 subtype cells co-expressing IFN-γ and GM-CSF were observed only in COVID-19 patients in the ICU, suggesting that pathogenic Th1 cells play an important role in the hyperinflammatory response to severe COVID-19 (77, 96).

As mentioned above, IFN plays an important role in the CD4^+^ T-cell-related immune reaction. CD4^+^ T cells, including Th1 and Th17 cells, are the predominant T-cell subsets in the BALF of sarcoidosis patients. Activated CD4^+^ T cells are characterized by the production of type II IFN (IFN-γ). Moreover, high levels of IFN-γ are common in COVID-19 patients because of granulomas formed in IFN-γ-activated macrophages. It is hypothesized that sarcoidal granulomas are associated with high IFN-γ and an imbalance of IFN (7). However, some researchers have found an impaired type I IFN response in severe COVID-19 patients, with no IFN-β and low IFN-α production (97, 98). The primary mechanism for limiting viral replication is type I interferon production (99). In contrast, viruses inhibit or delay the production and release of cytokines by altering cellular signaling pathways. Similarly, non-structural proteins (nsps) in SARS-CoV-2 can suppress type I IFN response and act as interferon antagonists (100). Mazzoni et al. (79) reported that an early dysregulated type I IFN response induced a series of immune response dysregulations, including cytokine storm, lymphocyte impairment, and the whole SARS-CoV-2 highly heterogeneous adaptive immune response.

### Tregs

With the development of flow cytometry, researchers have developed phenotypes of Treg cells. However, the developed phenotypes have presented conflicting results when analyzing the BALF and blood of sarcoidosis patients (101). d’Alessandro et al. (80) found that Treg cell percentage in the BALF of sarcoidosis patients was lower than their peripheral percentages. Furthermore, Treg cells in the blood of sarcoidosis patients showed increased numbers of activated and memory Tregs and decreased number of resting and naive Tregs. The phenotype CD45RO^+^ is predominant in late-stage memory Treg-derived cells in granulomas that lack CD7 and show elevated Treg levels of CD57 and KLRG-1 (102). Unlike classical Tregs, CD7-Treg cells have poor suppressive capacity and cannot limit immune responses. Instead, they amplify the immunoinflammatory response by secreting the inflammatory factor IL-4, which promotes the recruitment of mast cells and secretion of membrane-bound oncostatin M, inducing fibroblast proliferation and extracellular matrix protein synthesis in lung tissue (103).

Cytokine storms play a key role in the progression of severe respiratory diseases caused by viral infection. However, Treg cell activation may be associated with the prevention and treatment of cytokine storms (104). Tregs, in particular, can inhibit the release of TNF-α and cytokine IL-6 by producing IL-10 and TGF-β. The activation of Tregs in lung tissue is characterized by the release of IL-10, which suppresses excessive inflammatory responses (105), reflecting the severity of the COVID-19 inflammatory response (105). Loss of CD45RA^+^ Tregs and elevated levels of IL-10 have been observed in severe COVID-19 patients, which may lead to hyperimmunity and high mortality (106). In contrast, TGF-β1 produced by Treg can activate lung fibroblasts, and IL-10 activates alveolar macrophages, thereby inducing lung fibroblasts to extensively synthesize extracellular matrix substances (107). Thus, it is clear that COVID-19 patients develop granulomatous and/or pulmonary fibrosis in later stages. However, further verification is required to determine whether it is related to the excessive activation of Treg.

### Tfh Cells

Follicular helper T cells (Tfh) are a class of CD4^+^ T cells that participate in helper T-cell-dependent antibody responses and provide contact-dependent and cytokine signaling to B cells (108). In sarcoidosis, the Tfh cell population changes, with an increase in Tfh2 and Tfh17 cell count and a decrease in Tfh1 and Tfh17.1-like cell count (109). However, in COVID-19 convalescent patients, changes in the subsets of circulating Tfh (cTfh) were observed, with an increase in the number of cTfh1 and cTfh2 cells and a decrease in the number of cTfh17 cells (110). Experimental studies have shown that Tfh1 cells can trigger apoptosis in activated naive B cells. Although the role of Tfh cells in the development and progression of sarcoidosis is unclear, the predominant Tfh subpopulation appears to be associated with a naive phenotype with biased activation of B cells and hypergammaglobulinemia in sarcoidosis (111, 112). Meanwhile, a study found that the distribution of peripheral blood B-cell subsets in sarcoidosis patients was altered, with a reduced frequency of memory B-cell subsets and a predominance of “naive” and activated B-cell subsets (108). Tfh2 and Tfh17 activate and induce IgM, IgG, and IgA secretion in naive B cells by the production of the cytokine IL-21. These data suggest that B cells may be involved in the pathogenesis of sarcoidosis. Therefore, dysfunctional B-cell maturation disorders and changes in humoral immune responses in COVID-19 patients may be caused by impaired differentiation of Tfh-like cells. Golovkin et al. (113) analyzed Tfh-like cell subsets in COVID-19 patients and reported an imbalance of pro-inflammatory Tfh17-like cells that strongly correlate with disease severity. In addition, Tfh2/Tfh17 dominance has been observed in several other autoimmune diseases. Therefore, there is an underlying mechanism of sarcoid granulomas in SARS-CoV-2 convalescent patients associated with the impacts of Tfh.

### Cytokine Storm

Several changes in a series of cytokine levels have been observed in sarcoidosis and SARS-CoV-2. Along with extensive IFN-γ production in the lungs of sarcoidosis patients, the expression of cytokines, including pro-inflammatory cytokines, such as TNF, IL-12, IL-18, and IL-6, and regulatory cytokines, such as TGF-β and IL-10, has been reported to be upregulated in affected tissues (94). Moreover, higher levels of IL-2, IL-4, and IL-17A were observed in the serum of sarcoidosis patients than in the serum of healthy individuals (80). The cytokine storm associated with COVID-19 is a complex interconnected network involving both innate and adaptive immunity as well as multiple immune cells and cytokines. Specific manifestations are highly elevated clinical inflammatory biomarkers and correspondingly elevated serum cytokine levels, including IL-1β, IL-1RA, IL-2, IL-4, IL-6, IL-7, IL-10, IL-19, G-CSF, TNF-α, and IFN-γ (114–117). Dysregulated immune responses involving increased cytokine levels and immune system hyperactivation include a potentially life-threatening systemic inflammatory syndrome with consequent systemic inflammatory response syndrome (SIRS) and MODS (79).

However, the appearance of cytokine storm is only the manifestation of severe COVID-19 patients, with most of the other infected patients showing relatively minor cytokine changes, indicating mild immune reactions. Moreover, granulomatous manifestations were not observed in every mild patient. In the presence of unknown or self-antigens in the lungs, alveolar macrophages and dendritic cells are induced to recognize and take up the antigen. A study reported higher levels of mononuclear phagocytes (MNPs) in the blood and BALF of sarcoidosis patients than in healthy controls. An RNA-sequencing analysis showed the presence of highly inflammatory MNPs in the BALF of sarcoidosis patients, suggesting that pulmonary monocytes and monocyte-derived cells are highly inflammatory and may serve as predictors of disease outcome (118). CD4^+^ T cells exhibit a more aggressive Th1-dominant phenotype expressing T-bet and producing higher IFN-γ levels. However, they lack regulatory capacity and are unable to limit the immune reactions and even enhance the inflammatory reactions, resulting in tissue damage. B-cell anergy leads to decreased antigen specificity even with increased total Ig concentrations (119). In conclusion, although these immune responses are effective for antigen clearance, the lymphocytes generated against antigens have low activity and poor specificity. This process lasts for a long time and gradually turns into chronic inflammation, causing damage to lung tissue and the potential for permanent fibrotic scarring. Therefore, further research is required to determine the cause of nodular manifestations in COVID-19 patients.

For the treatment of COVID-19 with sarcoidal granulomas, the treatment of sarcoidosis can be referred. If there is progression in these patients, including progressive impairment of pulmonary function, a major radiographic progression (such as the development of cavities or fibrosis), glucocorticoids and immunosuppressants can be given after determining virus inactivity, or even cytokine monoclonal antibody therapy, such as the anti-TNF monoclonal antibody infliximab, is mainly used for the treatment of sarcoidosis as off-label third-line therapy (7). However, these patients may have sarcoidosis susceptibility genes. Therefore, it is recommended to avoid exposure to the susceptibility factors of sarcoidosis as much as possible in the future. Likewise, COVID-19 has given us some hints for the therapeutic strategies for sarcoidosis. For patients who have been diagnosed with sarcoidosis, it is necessary to carefully ask whether there is a history of viral infection before the onset of the disease, and if symptoms persist, antiviral therapy can be taken to avoid further imbalance of the immune response. In addition, further research should be conducted on the pathogenesis of sarcoidosis caused by viral infection to analyze immunoinflammatory dysregulation induced by infection.

## Cell Death Pathways

Microarray analysis of sarcoid cells and whole-exome sequencing of familial forms of sarcoidosis show unique transcriptional profiles with distinct autophagy and apoptosis mutation pathways. Meanwhile, SARS-CoV-2 takes advantage of particular autophagy signaling pathways by avoiding certain regulatory mechanisms to either escape or inhibit host cell defenses, thus helping the virus to undermine host cell antiviral immunity (120). This section discusses how COVID-19 and sarcoidosis may have some common cellular death pathways for the regulation of autophagy, apoptosis, and programmed cell death (PD-1/PD-L1 axis), which might help us better understand the strong association between SARS-CoV-2 and sarcoidosis (**Table 2**).

**Table 2:** The features of sarcoidosis and COVID-19 on cell death pathways.

	Sarcoidosis	COVID-19
Autophagy	Encoding autophagy genes: mTOR and Rac1 mutations	Using autophagy protein LC3 and Atg12 to escape host cell defenses
		Activate autophagy through the AMP kinase–mTOR pathway
Apoptosis	Inhibiting apoptosis: driving granuloma initiation and maintenance	Increased expression of pro-apoptotic genes in DC cells
		Upregulation of apoptosis-related genes in B cells
		Increased apoptotic T cells by expressing cleaved caspase-3 and/or cleaved PARP
PD-1/PD-L1 axis	The low expression of PD-1 could induce and maintain granuloma formation	T cells highly express PD-1 to diminish lymphocyte percentage
		Lower expression of PD-1 on T cells of long-time clinically recovered patients
		Higher expressions of PD-L1 with severe clinical infection in monocytes, DCs, and granulocytes

mTOR, mammalian target of rapamycin; Rac1, Ras-related C3 botulinum toxin substrate 1; PARP, poly ADP-ribose polymerase.

### Autophagy and Apoptosis

Autophagy and apoptosis are two parts of cell death pathways. Autophagy is a strictly programmed form of cell death mediated by multiple cellular signaling pathways and characterized by a highly controlled breakdown of cellular structures (121, 122).

Using whole-exome sequencing of familial sarcoidosis, researchers have identified genetic mutations in essential factors that regulate autophagy, such as the mammalian target of rapamycin (mTOR) and Rac1 molecular hubs, that result in constitutional defects in the regulation of macroautophagy. Furthermore, a recent study reported that granuloma formation in a Tsc2^−/−^ knockout mouse model was induced by the activation of the metabolic checkpoint kinase mTORC1 in macrophages (123). Further experiments demonstrated that mTORC1 induces granuloma initiation and maintenance by promoting metabolic reprogramming *via* cyclin-dependent kinase 4 (CDK4) while simultaneously inhibiting NF-κB signaling and apoptosis. Induction of apoptosis by inhibiting mTORC1 activity completely eliminated granulomas in bone marrow TSC2-deficient mice. Meanwhile, a clinical study (124) compared the genotypes of patients with self-limited and progressive active sarcoidosis by GSEA for hallmark gene sets and found that the mTORC1 pathway was significantly enriched in the progressive disease group, while mRNA expression of TSC1 was significantly decreased. These findings suggest that mTORC1-dependent macrophage proliferation may contribute to disease progression in sarcoidosis, and raise the hypothesis that activation of mTORC1 by antigens that cannot be cleared induces hypertrophy and granuloma formation. Considering the above findings, the occurrence and progression of sarcoidosis could be related to defects in the regulation of autophagy by mTORC1 pathways.

The autophagy process mainly includes autophagosome formation, fusion of the autophagosome with the lysosome, and proteolytic degradation of lysosomal proteases. Coronaviruses may evolve various strategies to escape host cell defense by exploiting the above autophagy processes. Preliminary studies observed that host cell autophagy proteins LC3 and Atg12 show co-localization with the coronavirus replicase protein nsp8, suggesting that the viral replication transcription complexes utilize components of cellular autophagy in the production of double-membrane vesicles (DMVs) (125). In addition, SARS-CoV-2 caused a marked decrease in ACE2 concentrations by increasing the number of receptors in host cells, resulting in increased levels of Ang II and its degradation products. Ang II massively stimulates apoptosis and is a negative regulator of autophagy (126). Moreover, molecular and bioinformatic studies have analyzed that the SARS-CoV-2 spike protein can also bind to cell surface receptors of glucose-regulated protein 78 or heat shock 70 kD protein 5, which activates autophagy through the AMP kinase–mTOR pathway (127).

Likewise, it has been reported that HCoV infection can induce apoptosis. Apoptosis features have also been observed in the autopsies of samples from the respiratory tract, lungs, heart, and liver of SARS-CoV-2-infected individuals (128). Multiple cellular signaling pathways can induce apoptosis in SARS-CoV-2-infected cells. Elevated levels of circulating apoptotic T cells expressing cleaved caspase-3 and/or cleaved caspase-3 poly ADP-ribose polymerase (PARP) were observed in COVID-19 patients (129). Apoptosis-related genes are upregulated in T cells and B cells of severely infected patients, suggesting that their reduced numbers may be related. Similarly, the expression of pro-apoptotic genes was also upregulated in dendritic cells (DCs) of COVID-19 patients (130). Considering these findings, it can be concluded that upregulated apoptotic gene expression in immune cells and increased cell death may explain the lymphopenia observed in COVID-19 patients, which in turn suppresses the host immune response.

In summary, it is clear that COVID-19 can induce changes in autophagy pathways of the host cells and cause apoptosis, allowing it to easily invade host cells. At the same time, the autophagy pathway of sarcoidosis patients is actually defective. Therefore, we hypothesize that defected autophagy pathways in sarcoidosis patients may make it easier for SARS-CoV-2 to invade the host cell and cause infection, or the existence of defected autophagy may make COVID-19 symptoms less visible. On the other hand, changes in the autophagy system induce sarcoidosis-like granulomas in SARS-Cov-2-infected patients.

### PD-1/PD-L1 Axis

In the immune system, PD-1 acts as an immune checkpoint protein to control immunoinflammatory responses by regulating programmed cell death signaling pathways, particularly by increasing the apoptosis of specific T cells and reducing the apoptosis of regulatory T cells (mainly including suppressor T cells and anti-inflammatory effects) (131). Previous studies have shown that the PD-1/PD-L1 axis, a member of the immunoglobulin superfamily, can control immunoinflammatory responses by regulating the magnitude and quality of T-cell responses and plays an important role in inducing and maintaining central and peripheral immunity tolerance (132).

SARS-CoV-2-associated reactive T cells exhibit increased PD-1 expression, indicating that they have been recently activated (133). This characteristic is more evident in symptomatic patients than in asymptomatic patients. A decrease in the percentage of lymphocytes in COVID-19 patients indicates a trend of severe disease. Elevated levels of IL-6, IL-10, and TNF-α in COVID-19 patients promote the expression of PD-1 and T-cell immunoglobulin mucin 3 (Tim-3) on the surface of peripheral T cells that act as exhaustion signals, resulting in decreased T-cell function and memory T-cell activity. In addition, PD-L1 expression in monocytes, DCs, and granulocytes was higher in patients with severe clinical infection than in patients with mild infection. Moreover, PD-L1 upregulation was observed in the biopsies of SARS-CoV-2-infected cells and lung samples, and elevated serum soluble PD-L1 levels are considered a negative prognostic marker (134).

PD-1 is related to immune cell numbers as well as T-cell functions, resulting in decreased immune cell reactivity. The binding of PD-L1 on the surface of monocytes and the expression of PD-1 on the surface of CD8^+^ T lymphocytes limit their antiviral ability and lead to disease progression (135). Therefore, inhibition of the PD-1/PD-L1 axis may increase T-cell activity, enhance the host’s ability to remove and clear viral particles, and eliminate viral infection. Thus, SARS-CoV-2 infection can alter the adaptive immune system and even innate immunity.

As mentioned earlier, the pathogenesis of autoimmune diseases involves a disturbed balance between the activation and regulation of immune responses. A study reported several markers that may play a key role in the pathogenesis of sarcoidosis, most prominently, increased expression of regulatory receptors CTLA-4 and PD-1, as well as inducible co-stimulator (ICOS) in the CD4^+^ T-cell population in patients with nodular LS (136). The limited PD-1 expression in LS patients with favorable prognosis suggests that the balance between immune activation and regulation is essential for the development of granulomatous disease. The low expression of PD-1 could induce and maintain granuloma formation. Some studies suggest that increased PD-1 expression correlates with decreased CD4^+^ T-cell proliferation and contributes to the pathogenicity of sarcoidosis upon infection (137, 138). However, because sarcoidosis is an autoimmune disease, it is suggested that PD-1 is upregulated to control the adaptive immune response to persistent tissue (self) antigens that the patient cannot clear. On the one hand, loss of PD-1 results in accelerated viral clearance, whereas downregulation of PD-1 may lead to severe tissue damage (139). These results suggest that the PD-1/PD-L1 axis helps to modulate the intensity and quality of active attack by the immune system, balance antigen elimination and excessive inflammatory damage, and prevent excessive tissue damage.

Compared with sarcoidosis, increased expression of PD-1 was observed in SARS-CoV-2-infected patients at the early stages of infection, particularly in severe patients. Phenotypic analysis showed lower levels of PD-1 expression in CD4^+^ T cells of long-time COVID-19 clinically recovered patients than in those of healthy controls. Therefore, granulomatous responses in COVID-19 patients may act as a marker of recovery rather than acute infection (140). Moreover, sarcoid-like granulomas were also observed in patients treated with IFN-α, again supporting the hypothesis. This phenomenon has also been reported in patients receiving anti-PD-1 therapy (e.g., metastatic melanoma) (141), suggesting that inhibition of these immunosuppressive functions results in granuloma formation (7). Further investigations are required to better understand the relationship between the appearance of sarcoid-like nodules in COVID-19 patients and the expression level of PD-1.

## Conclusion

In this study, we analyzed the possible connections between COVID-19 and sarcoidosis, including the role of the RAS in the respiratory system, immune response, and cell death pathways. By reviewing all the above aspects, this study aimed to determine whether patients with sarcoidosis are susceptible to SARS-CoV-2 and to explore the mechanism of granuloma formation in SARS-CoV-2-infected patients to provide us with new prospects for the treatment of COVID-19. This review can help us better understand the pathogenesis of sarcoidosis and provide us with a deeper understanding of the diagnosis and treatment of sarcoidosis.

## Author Contributions

All authors listed have made a substantial, direct, and intellectual contribution to the work and approved it for publication.

## Funding

This study was supported by the Technology Research Funds of Jilin Province (20190303162SF), the Medical and Health Project Funds of Jilin Province (20200708083YY, 2020SCZT019, 20191102012YY), and the Disciplinary Crossing and Integration and Innovation Cultivation Project of Jilin University (JLUXKJC2020212).

## Conflict of Interest

The authors declare that the research was conducted in the absence of any commercial or financial relationships that could be construed as a potential conflict of interest.

## Publisher’s Note

All claims expressed in this article are solely those of the authors and do not necessarily represent those of their affiliated organizations, or those of the publisher, the editors and the reviewers. Any product that may be evaluated in this article, or claim that may be made by its manufacturer, is not guaranteed or endorsed by the publisher.
